# Existence of Multiple ESBL Genes among Phenotypically Confirmed ESBL Producing *Klebsiella pneumoniae* and *Escherichia coli* Concurrently Isolated from Clinical, Colonization and Contamination Samples from Neonatal Units at Bugando Medical Center, Mwanza, Tanzania

**DOI:** 10.3390/antibiotics10050476

**Published:** 2021-04-21

**Authors:** Vitus Silago, Dory Kovacs, Happyness Samson, Jeremiah Seni, Louise Matthews, Katarina Oravcová, Athumani M. Lupindu, Abubakar S. Hoza, Stephen E. Mshana

**Affiliations:** 1 Department of Veterinary Microbiology, Parasitology and Biotechnology, College of Veterinary Medicine and Biomedical Sciences, Sokoine University of Agriculture, P.O. Box 3000, Morogoro 67125, Tanzania; abhoza@sua.ac.tz; 2 Department of Microbiology and Immunology, Weill Bugando School of Medicine, Catholic University of Health and Allied Sciences, P.O. Box 1464, Bugando, Mwanza 33109, Tanzania; hsamson@bugando.ac.tz (H.S.); senijj80@bugando.ac.tz (J.S.); mshana72@bugando.ac.tz (S.E.M.); 3 Institute of Biodiversity, Animal Health and Comparative Medicine, University of Glasgow, Glasgow G12 8QQ, UK; d.kovacs.1@research.gla.ac.uk (D.K.); Louise.Matthews@glasgow.ac.uk (L.M.); Katarina.Oravcova@glasgow.ac.uk (K.O.); 4 Department of Veterinary Medicine and Public Health, College of Veterinary Medicine and Biomedical Sciences, Sokoine University of Agriculture, P.O. Box 3021, Morogoro 67125, Tanzania; alupindu@sua.ac.tz

**Keywords:** antimicrobial resistance, extended-spectrum beta-lactamase, *Escherichia coli*, *Klebsiella pneumoniae*

## Abstract

The proportions and similarities of extended-spectrum β-lactamase (ESBL) producing *K. pneumoniae* (ESBL-KP) and *E. coli* (ESBL-EC) carrying multiple ESBL genes is poorly known at our setting. This study investigated the existence of multiple ESBL genes (*bla*_CTX-M_, *bla*_TEM,_ and *bla*_SHV_) among ESBL-KP and ESBL-EC concurrently isolated from clinical, colonization, and contamination samples from neonatology units in Mwanza-Tanzania. Twenty and 55 presumptive ESBL-EC and ESBL-KP, respectively, from a previous study archived at −80 °C were successfully recovered for this study. Isolates were screened and confirmed for production of ESBLs by phenotypic methods followed by multiplex PCR assay to determine ESBL genes. All (100%) and 97.3% of presumptive ESBL isolates were phenotypically confirmed by Clinical and Laboratory Standards Institute (CLSI) and modified double-disc synergy methods, respectively. About 93.3% (70/75) of phenotypically confirmed ESBL isolates had at least one ESBL gene, whereby for 62.9% (44/70), all ESBL genes (*bla*_CTX-M_, *bla*_TEM,_ and *bla*_SHV_) were detected. Eight pairs of ESBL bacteria show similar patterns of antibiotics susceptibility and ESBL genes. ESBL-KP and ESBL-EC, concurrently isolated from clinical, colonization and contamination samples, harbored multiple ESBL genes. Further, eight pairs of ESBL isolates had similar patterns of antibiotics susceptibility and ESBL genes, suggesting transmission of and/or sharing of mobile genetic elements (MGEs) among ESBL-KP and ESBL-EC.

## 1. Introduction

Extended-spectrum β-lactamases (ESBLs) are enzymes which hydrolyze the beta-lactam ring of β-lactam antibiotics conferring bacterial resistance to penicillins (e.g., ampicillin and amoxicillin); first- (e.g., cephalexin), second- (e.g., cefuroxime) and third- (e.g., ceftriaxone and cefotaxime) generation cephalosporins; and monobactams (e.g., aztreonam) [1,2]. Therefore, ESBL producing bacteria respond poorly to β-lactams, leading to higher medical costs, prolonged hospital stays, loss of prophylactic protection, and increased mortality [3,4]. ESBLs are not effective in hydrolyzing cephamycins (e.g., cefoxitin and cefotetan) and carbapenems (e.g., imipenem and meropenem), therefore, these antibiotic agents—notably carbapenems, are recommended for the treatment of infections due to ESBL producers [5,6].

ESBL enzymes are mediated by genes, commonly *bla*_CTX-M_, *bla*_TEM,_ and *bla*_SHV_, which are harbored by self-transmissible conjugative plasmids that are horizontally shared within the same and different species of bacteria [7,8]. ESBLs production is inhibited by beta-lactamase inhibitors such as clavulanic acid and tazobactam, a trait that can be used for phenotypic detection of ESBL production among Enterobacteriaceae, particularly *E. coli*, *K. pneumoniae*, *K. oxytoca*, and *P. mirabilis* [9,10]. Molecular-based methods, i.e., multiplex PCR assays, are more useful for ESBL testing for epidemiological comparison or infection control purposes [11].

The proportions of ESBL producing Gram-negative bacteria (ESBL-GNB) causing infections have been found to range from 10.5% to 50.3% in Mwanza, Tanzania [12,13]. The *bla*_CTX-M_ gene has been widely studied among ESBL genes and predominantly has been reported to range from 76% to 95% among Gram-negative bacteria isolated from clinical and environmental samples in this region [14,15,16]. Despite the presence of the above information, the proportions (by multiplex PCR assay) and similarities (similar patterns of antibiotics susceptibility and ESBL genes) of extended-spectrum β-lactamase (ESBL) producing *K. pneumoniae* (ESBL-KP) and *E. coli* (ESBL-EC) carrying multiple ESBL genes (*bla*_CTX-M_, *bla*_TEM,_ and *bla*_SHV_) is poorly understood at our setting. This study investigated the existence of ESBL genes (*bla*_CTX-M_, *bla*_TEM,_ and *bla*_SHV_) by multiplex PCR assay among phenotypically confirmed ESBL-KP and ESBL-EC concurrently isolated from clinical, colonization and contamination samples. We also investigated pairs of ESBL isolates from different sources with similar patterns of antibiotics susceptibility and ESBL genes. The results suggest the possible cross-transmission of ESBL isolates and/or sharing of mobile genetics elements (MGEs) among *E. coli* and/or *K. pneumoniae* in NICU and neonatology unit at Bugando Medical Centre (BMC), Mwanza-Tanzania.

## 2. Results

### 2.1. Phenotypic Screening and Confirmation of ESBL Production

**Figure 1:**
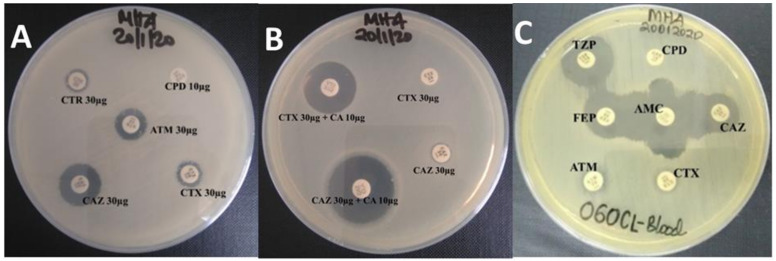
Phenotypic screening and confirmation of extended-spectrum β-lactamases (ESBL) production. (**A**): showing a positive screening test by CLSI screening method for ESBL production using 3rd-generation cephalosporins and aztreonam discs; (**B**): showing phenotypically confirmed ESBL producer by CLSI combination disc method for phenotypic confirmation of ESBL production from *K. pneumoniae*, *K. oxytoca*, *E. coli,* and *P. mirabilis*; and (**C**): showing phenotypically confirmed ESBL producer by modified double-disc synergy test.

All isolates (100%, *n* = 75) were positive for ESBL production by the CLSI screening method and were also phenotypically confirmed to be ESBL producers by CLSI-combined disc detection (CLSI-CDD) method. Two presumptive ESBL-KP (one isolated from neonate’s blood and another isolated from neonate’s cot swab) were negative for ESBL production by the modified double disc synergy (MDDS) method (Figure 1).

### 2.2. Multiple ESBL Genes Harbored by Phenotypically Confirmed ESBL-KP and ESBL-EC

**Figure 2:**
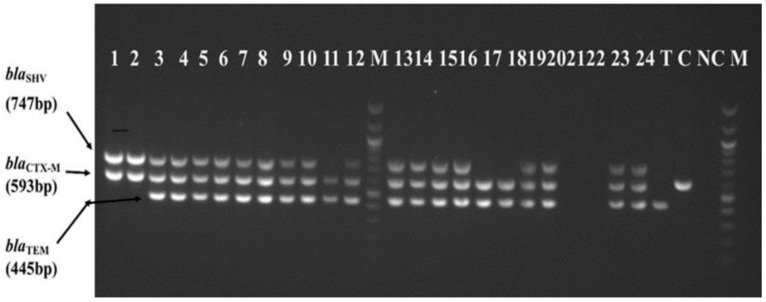
Results of multiplex PCR amplification of genes bla_SHV_ (747 bp), bla_CTX-M_ (593 bp) and bla_TEM_ (445 bp) Lanes: 1 to 12 = DNA samples of phenotypic confirmed ESBL isolates; M = 100 bp DNA ladder; 13 to 24 = DNA samples of phenotypic confirmed ESBL isolates; T = bla_TEM_ positive control; C = bla_CTX-M_ positive control; NC = negative control; and M = 100 bp DNA ladder.

**Figure 3:**
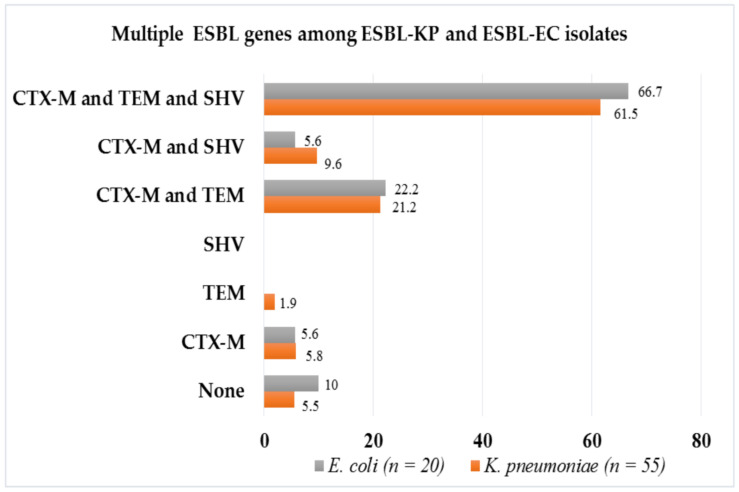
Existence and proportions of multiple ESBL genes among phenotypic confirmed ESBL-KP and ESBL-EC isolates.

By multiplex PCR assay, about 93.3% (*n* = 70) of phenotypically confirmed ESBL bacteria tested had at least one ESBL gene (*bla*_CTX-M_/*bla*_TEM_/*bla*_SHV_), of which the majority harbored *bla*_CTX-M_ (98.6%, *n* = 69) followed by *bla*_TEM_ (85.7%, *n* = 60) and *bla*_SHV_ (71.4%, *n* = 50). The combinations of ESBL genes observed were *bla*_CTX-M_/*bla*_TEM_/*bla*_SHV_ (62.9%, *n* = 44), *bla*_CTX-M_/*bla*_TEM_ (21.4%, *n* = 15) and *bla*_CTX-M_/*bla*_SHV_ (8.6%, *n* = 6), as shown in Figure 2 below. Specifically, about 94.5% (52/55) and 90% (18/20) of phenotypically confirmed ESBL-KP and ESBL-EC had at least one ESBL gene, respectively (Figure 3).

### 2.3. Combination of ESBL Genes in Association with Resistance to None β-Lactam Antibiotics and Meropenem

Over 50% of ESBL isolates (ESBL-KP and ESBL-EC) harboring all three ESBL genes (*bla*_CTX-M_/*bla*_TEM_/*bla*_SHV_) exhibited resistance towards SXT 25 µg (58.7%), CN 30 µg (59.3%), CIP 5 µg (51.7%), and MEM 10 µg (100%) as shown in Table 1 below.

### 2.4. Pairs of ESBL Isolates Showing Similar Patterns of Antibiotics Susceptibility and ESBL Genes

Eight pairs of ESBL isolates exhibited similar patterns of antibiotics susceptibility and ESBL genes. Four pairs of ESBL-KP (ID: 275CL, 282CL, 285CL, and 387CL) paired between four neonate’s blood samples and rectal swabs; two pairs, one between ESBL-EC (ID: 231CL) from rectal swab and ESBL-KP (ID: 249CL) from cot swab of different neonates and another between ESBL-EC (ID: 249CL) from rectal swab and ESBL-KP (ID: 249CL) from cot swab of the same neonate; one pair between ESBL-KP (ID: 070CL) from neonate’s rectal swab and ESBL-KP (ID: CL053) from hand swab of healthcare worker (HCW); and another pair of ESBL-KP (ID: 249CL and 250CL) isolated from blood samples of two different neonates who shared a cot (Table 2).

## 3. Discussion

This study confirmed that the *bla*_CTX-M_, *bla*_TEM_, and *bla*_SHV_ are the most common ESBL genes, with members of the *bla*_CTX-M_ family being particularly well disseminated and reported from all over the world [17,18,19]. In this study, we report the existence of multiple ESBL genes (*bla*_CTX-M_, *bla*_TEM_, and *bla*_SHV_) by multiplex PCR assay and similar patterns of antibiotics susceptibility and ESBL genes among phenotypically confirmed ESBL-KP and ESBL-EC. All isolates were concurrently isolated from clinical, colonization, and contamination samples from NICU and neonatology unit at Bugando Medical Centre (BMC). These findings suggest the possible cross-transmission of ESBL-KP and ESBL-EC and/or sharing of MGEs such as conjugative plasmids among *E. coli* and/or *K. pneumoniae* in this facility.

Phenotypically, 100% and 97.3% of presumptive ESBL-KP and ESBL-EC were confirmed for ESBL production by CLSI combination disc diffusion (CLSI-CDD) method [10] and modified double-disc synergy (MDDS) by Diab et al. [20], respectively. The CLSI-CDD method used both ceftazidime (CAZ 30 µg) and cefotaxime (CTX 30 µg) with and without clavulanic acid (CA 10 µg), respectively. This combination has been found to increase sensitivity in the detection of ESBL production among *K. pneumoniae*, *K. oxytoca*, *E. coli,* and *P. mirabilis* [21]. All presumptive ESBL-KP and ESBL-EC in our study were confirmed using this method. The MDDS method failed to confirm ESBL production in two ESBL-KP isolated from neonate’s blood and cot. The two ESBL-KP may be co-producing other β-lactamase enzymes such as AmpC β-lactamase, carbapenemase-producing *K. pneumoniae* (KPC), or Metallo-β-lactamase (MBL). Co-production of ESBL and AmpC β-lactamase or KPC or MBL lowers the sensitivity of synergism-based methods in the detection of ESBL production [22]. Although synergism-based methods remain feasible for the detection of ESBL production notable in *E. coli* and *K. pneumoniae* in most low- and middle-income countries (LMICs).

In the current study, we observed that ESBL-KP and ESBL-EC isolated from different sources (blood, rectal swabs, cot swabs, and hand swabs) harbor multiple ESBL genes (*bla*_CTX-M_, *bla*_TEM_, and *bla*_SHV_), similar to a study from India [23]. However, our findings are contrary to a study done in another region of Tanzania by Ndugulile et al., who reported less occurrence of multiple ESBL genes among ESBL producing Gram-negative bacteria causing nosocomial infections in ICUs [24]. We also observed that ESBL-KP and ESBL-EC harboring all three genes (*bla*_CTX-M_, *bla*_TEM_, and *bla*_SHV_) were more resistant to trimethoprim-sulfamethoxazole, gentamicin, ciprofloxacin, and meropenem. This might be due to the fact that the same conjugative plasmids harboring ESBL genes, also often harbor other antimicrobial resistance genes (ARGs) of other antibiotic categories such as aminoglycosides (e.g., gentamicin), trimethoprim, sulfamethoxazole (e.g., trimethoprim-sulfamethoxazole), and fluoroquinolones (e.g., ciprofloxacin) [25,26].

Out of the three ESBL genes (*bla*_CTX-M_, *bla*_TEM_, and *bla*_SHV_) analyzed, the *bla*_CTX-M_ was predominant (98.6%). The predominance of *bla*_CTX-M_ is in line with other studies from the same region and elsewhere, despite the difference in the detection methods used [14,15,27,28]. Members of the CTX-M family notably *bla*_CTX-M-15_ are commonly encoded by conjugative epidemic plasmids, i.e., IncFII which plays an important role in their successful dissemination and, therefore, their predominance [28,29,30]. Five (6.7%; 3 ESBL-KP; and 2 ESBL-EC) out of 75 phenotypically confirmed ESBL-KP (*n* = 55) and ESBL-EC (*n* = 20) were negative for any of the ESBL genes tested. These negative isolates might be harboring other ESBL genes such as *bla*_OXA_, *bla*_PER_, *bla*_VEB_, and *bla*_BEL_ [7,31], which were not tested in this study.

This study found eight pairs (4 pairs of blood samples vs. rectal swabs; 2 pairs of rectal swabs vs. cot swabs; 1 pair of blood sample vs. blood sample; 1 pair of rectal swab and hand swab of HCW) of ESBL isolates having similar patterns of antibiotics susceptibility and ESBL genes. This observation suggests that ESBL-KP and ESBL-EC colonizing the guts of patients, contaminating patients’ immediate environments, infecting patients, and contaminating the hands of HCWs are a potential threat and may be a potential source of infection. Our results suggest the possible transmission of, and/or sharing of, MGEs, e.g., conjugative plasmids harboring ARGs among ESB-KP and ESBL-EC in our setting. Pairs of similar ESBL isolates from blood and rectal samples of the same neonates suggest possible microbial translocation; the same bacteria carried in the rectum is also isolated from the own blood sample [32,33]. Pairs of similar ESBL isolates from rectal and cot samples suggest that colonized patients are shedding the ESBL bacteria colonizing their guts, which significantly contaminates their immediate environments [34]. A pair of ESBL-KP isolated from blood samples of two neonates who shared a cot suggests patient-to-patient transmission. A pair of ESBL-KP isolated from neonate’s rectal swab and hand swab of HCW suggests that the hands of HCWs become contaminated with ESBL pathogens when attending patients. Thus, contaminated hands of HCWs are potential vehicles of transmitting ESBL pathogens between one patient and another and/or patients and their immediate environments [35]. Generally, these findings suggest the possibilities of cross-transmission of ESBL-KP and ESBL-EC and/or sharing of MGEs such as conjugative plasmids harboring ESBL genes among *K. pneumoniae* and *E. coli* at this setting.

In conclusion, our findings highlight the existence of multiple ESBL genes among phenotypically confirmed ESBL-KP and ESBL-EC isolated from clinical, colonization, and contamination samples in our setting. ESBL isolates harboring all the three ESBL genes exhibited more resistance to other antibiotics belonging in non-β-lactam classes. Our findings also suggest possibilities of cross-transmission of ESBL-KP and ESBL-EC and/or sharing of MGEs among *K. pneumoniae* and *E. coli* at this facility. To the best of our knowledge, this is the first study to isolate ESBL-KP harboring *bla*_CTX-M_ and *bla*_TEM_ from the contaminated hands of HCW. Further, it highlights the potential role of contaminated patients’ environment and hands of HCWs in the transmission of ESBL producing pathogens in this setting. We, therefore recommend improved cleaning and decontamination of inanimate hospital surfaces (e.g., patients’ beds and cots) and observation of standard precaution, i.e., hand hygiene among HCWs before and after touching patients and patients’ immediate environments.

## 4. Materials and Methods

### 4.1. Study Design, Duration, Setting and Population

This cross-sectional study was conducted in January 2020 at the Catholic University of Health and Allied Sciences (CUHAS) in Mwanza, Tanzania. This study involved 75 presumptive ESBL producing bacteria, showing resistance to third-generation cephalosporins (3GCs), i.e., ceftriaxone and/or ceftazidime, recovered from a previous study [36]. The isolates were identified to species level by physiological and biochemical in-house-prepared tests, as reported in Koneman’s Color Atlas and Textbook of Diagnostic Microbiology, 7th Edition. In the current study, we included presumptive ESBL-KP and ESBL-EC concurrently isolated from neonates’ blood, rectal colonization, and cots’ contamination, and from contaminated hands of neonates’ mothers and healthcare workers (HCWs). Twenty presumptive ESBL-EC originated from neonates’ blood samples (*n* = 9), neonates’ rectal swab samples (*n* = 10) and neonate mother hand swab sample (*n* = 1); and 55 presumptive ESBL-KP originated from hand swab samples of HCWs (*n* = 2), neonates’ cot swab samples (*n* = 5), neonates’ blood samples (*n* = 29), neonates’ mothers hand swab samples (*n* = 3) and neonates’ rectal swab samples (*n* = 16) were recovered for this study.

### 4.2. Isolates Recovery and Phenotypic Screening of ESBL Production

Archived isolates (at −80 °C) were recovered by sub-culturing on MacConkey agar (MCA; Oxoid, Basingstoke, UK) plates which were incubated aerobically at 37 °C for 24 h. After incubation, a single colony from each plate of MCA was used for phenotypic screening of ESBL and AmpC β-lactamase productions as reported by Clinical and Laboratory Standards Institute (CLSI) [21] and Polsfuss et al. [37], respectively. Briefly, each test bacterium was suspended in sterile 0.85% normal saline and calibrated to 0.5 McFarland turbidity standard, and then swabbed on the entire surface of Muller Hinton agar (MHA; Oxoid, Basingstoke, UK) plate to make even lawns. Within 15 min, the following antibiotic discs (Oxoid, UK)—ceftriaxone 30 µg (CTR), ceftazidime 30 µg (CAZ), cefotaxime 30 µg (CTX), aztreonam 30 µg (ATM), and cefpodoxime 10 µg (CPD)—were seeded. MHA plates were incubated aerobically at 37 °C for 18 h. An isolate exhibiting resistance to CTR (≤25 mm), CAZ (≤22 mm), CTX (≤27 mm), ATM (≤27 mm), and CPD (≤17 mm) was considered a presumptive ESBL producer.

### 4.3. Phenotypic Confirmation of ESBL Production

#### 4.3.1. CLSI Combination Disc Diffusion (CLSI-CDD) Method

A combination disc diffusion (CDD) method, as recommended by CLSI [21] for phenotypic detection of ESBL production in *E. coli*, *K. pneumoniae*, *K. oxytoca,* and *P. mirabilis,* was used. Briefly, a lawn of test bacteria suspension equivalent to 0.5 McFarland turbidity standard solution was swabbed on surfaces of MHA plates, and then CAZ 30 µg and CTX 30 µg discs (Oxoid, UK) with and without clavulanic acid 10 µg (CA 10 µg) were seeded within 15 min. All plates were then incubated aerobically at 37 °C for 18 h. An isolate was phenotypically confirmed as an ESBL producer when a zone diameter difference of ≥5 mm was observed between both antibiotic discs with clavulanic acid and a similar agent without clavulanic acid.

#### 4.3.2. Modified Double Disc Synergy (MDDS) Method

A modified double-disc synergy (MDDS) test by Diab et al. [20] was used for further phenotypic confirmation of ESBL production. Briefly, lawns of test bacteria suspensions equivalent to 0.5 McFarland turbidity standard solution were swabbed on surfaces of MHA plates, and then antibiotic discs (Oxoid, UK): CAZ 30 µg, CTX 30 µg, CPD 10 µg, and FEP 30 µg were seeded side-by-side with amoxicillin-clavulanic acid 30 µg (AMC) at a distance of 15 mm. Another antibiotic disc (Oxoid, UK) of piperacillin-tazobactam 110 µg (TZP 110 µg) was also seeded on the same MHA plate side-by-side with FEP 30 µg at a distance of 22 mm. Enhanced zone of inhibition of CAZ 30 µg or CTX 30 µg or CPD 10 µg or FEP 30 µg towards AMC 30/10 µg and/or enhanced zone of inhibition of FEP 30 µg towards TZP 110 µg, phenotypically confirmed for ESBL production.

### 4.4. Antibiotic Susceptibility Testing (AST)

For antibiotics susceptibility testing (AST), zone diameters for trimethoprim-sulfamethoxazole 25 µg (SXT 25 µg), gentamicin 30 µg (CN 30 µg), ciprofloxacin 5 µg (CIP 5 µg), and meropenem 10 µg (MEM 10 µg) were retrieved from laboratory database of the previous study [36] and interpreted according to CLSI 2020 guidelines [21]. These antibiotics were selected to determine the ability of ESBL-KP and ESBL-EC to resist no β-lactam antibiotics except meropenem and to evaluate the existence of similar phenotypes among ESBL-KP and/or ESBL-EC.

### 4.5. Molecular Characterization of ESBL Genes

#### 4.5.1. DNA Extraction

A loopful (1 µL loop) of overnight growth of each isolate grown on an MCA plate was suspended in 500 µL of sterile, nuclease-free ultrapure water for DNA extraction. Suspensions were then centrifuged at 10,000 rpm for 10 min. The supernatant was discarded and pellets were re-suspended by adding 180 µL of buffer ATL (a tissue lysis buffer). DNA was extracted using QIAmp^®^ DNA Mini kit (QIAGEN, Hilden, Germany) following the manufacturer’s instructions from Gram-negative bacteria. The extracted DNA samples were quantified by dsDNA BR Kit on a Qubit 4 Fluorometer (ThermoFisher Scientific, Alexandra, Singapore) and checked for purity by gel electrophoresis using 1% agarose gel (ThermoFisher Scientific, Alexandra, Singapore). DNA samples were stored immediately at −20 °C until further processing.

#### 4.5.2. Multiplex PCR Amplification

Multiplex PCR assay by Monstein et al. [38] was used for detection of ESBL genes: *bla*_CTX-M_, *bla*_SHV_, and *bla*_TEM_ in DNA samples of ESBL-KP and ESBL-EC. Briefly, 2 µL of each DNA sample was amplified in a 25 µL reaction PCR Eppendorf tube containing HotStarTaq^®^ DNA polymerase master mix (QIAGEN, Hilden, Germany) and each primer at a reaction concentration of 200 nM. PCR reactions were carried out in thermal cycler machine (T100™, BIO-RAD, Kaki-Bukit, Singapore) which was conditioned at: initial denaturation at 95 °C for 5 min; 30 cycles of denaturation at 94 °C for 30 s, annealing at 56 °C for 30 s, and extension at 72 °C for 1 min; and a final extension at 72 °C for 10 min. PCR amplicons were separated electrophoretically on 1% agarose gel with SYBR Safe dye and visualized under UV light. For quality control of multiplex PCR assay, internal quality control was performed by running each DNA sample in duplicates and for external quality control, known control organisms harboring *bla*_CTX-M_, *bla*_TEM_, and *bla*_SHV_ were included in each run from DNA extraction to detection of ESBL genes.

### 4.6. Quality Control

ESBL-KP ATCC 700603 and ESBL-EC NCTC 13353 were used as control organisms.

### 4.7. Statistical Analysis

STATA software version 13.0 was used for data analysis. Data were presented as percentages and fractions. Chi-square test was used to show the association between a combination of ESBL genes and percentage resistance to SXT 25 µg, CN 30 µg, CIP 5 µg, and MEM 10 µg. A *p*-value less than 0.05 at a 95% confidence interval was used as a cut-off for statistical significance. Isolates exhibiting similar profiles of antibiotic susceptibility patterns and harboring similar ESBL genes were considered similar (for same species) or shared similar MGEs, conferring similar resistance patterns (for different species).

## Figures and Tables

**Table 1:** Combination of ESBL genes in association with resistance to none β-lactam antibiotics and meropenem.

Antibiotic Agent	Patterns of ESBL Genes Combinations					
	None	CTX-M or TEM	CTX-M and TEM	CTX-M and SHV	CTX-M, TEM and SHV	*p* Value
SXT (*n* = 75)	5 (6.7%)	5 (6.7%)	15 (20%)	6 (8.0%)	44 (58.7%)	-
CN (*n* = 59)	4 (6.8%)	5 (8.5%)	10 (16.9%)	5 (8.5%)	35 (59.3%)	0.601
CIP (*n* = 26)	2 (6.7%)	2 (6.7%)	7 (24.1%)	3 (10.3%)	15 (51.7%)	0.836
MEM (*n* = 4)	-	-	-	-	4 (100%)	0.562

Key: SXT = trimethoprim-sulfamethoxazole; CN = gentamicin; CIP = ciprofloxacin; and MEM = meropenem.

**Table 2:** ESBL isolates exhibiting similar profiles of antibiotic susceptibility patterns and ESBL genes.

ID	Isolate	Source	MDDS	SXT	CN	CIP	MEM	Presence of *bla* Alleles
070CL CL053	*K. pneumoniae*	rectal swab	positive	6 (R)	8 (R)	32 (S)	30 (S)	CTX-M + TEM
	*K. pneumoniae*	HCW hand	positive	6 (R)	6 (R)	32 (S)	34 (S)	CTX-M + TEM
231CL 249CL	*E. coli*	rectal swab	positive	6 (R)	14 (I)	30 (S)	30 (S)	CTX-M + TEM + SHV
	*K. pneumoniae*	cot	positive	6 (R)	14 (I)	30 (S)	29 (S)	CTX-M + TEM + SHV
249CL	*E. coli*	rectal swab	positive	6 (R)	14 (I)	28 (S)	28 (S)	CTX-M + TEM + SHV
	*K. pneumoniae*	cot	positive	6 (R)	14 (I)	30 (S)	29 (S)	CTX-M + TEM + SHV
249CL 250CL	*K. pneumoniae*	blood	positive	6 (R)	14 (I)	28 (S)	12 (R)	CTX-M + TEM + SHV
	*K. pneumoniae*	blood	positive	6 (R)	14 (I)	27 (S)	11 (R)	CTX-M + TEM + SHV
275CL	*K. pneumoniae*	blood	positive	6 (R)	6 (R)	17 (I)	28 (S)	CTX-M + TEM
	*K. pneumoniae*	rectal swab	positive	6 (R)	8 (R)	20 (I)	32 (S)	CTX-M + TEM
282CL	*K. pneumoniae*	blood	positive	6 (R)	6 (R)	6 (R)	30 (S)	CTX-M + TEM + SHV
	*K. pneumoniae*	rectal swab	positive	6 (R)	6 (R)	6 (R)	30 (S)	CTX-M + TEM + SHV
285CL	*K. pneumoniae*	blood	positive	12(R)	6 (R)	30 (S)	30 (S)	CTX-M + TEM + SHV
	*K. pneumoniae*	rectal swab	positive	6 (R)	6 (R)	28 (S)	30 (S)	CTX-M + TEM + SHV
387CL	*K. pneumoniae*	blood	positive	6 (R)	6 (R)	19 (I)	30 (S)	CTX-M + TEM + SHV
	*K. pneumoniae*	rectal swab	positive	6 (R)	6 (R)	15 (R)	30 (S)	CTX-M + TEM + SHV

Notes: ID = identification; MDDS = modified double disc synergy; SXT = trimethoprim-sulfamethoxazole; CN = gentamicin; CIP = ciprofloxacin; MEM = meropenem; HCW = healthcare worker; I = intermediate; R = resistant; and S = sensitive.

## Data Availability

The data presented in this study are available on request from the corresponding author. The data are not publicly available due to privacy restrictions.
